# Concomitant Mediterranean spotted fever and systemic lupus erythematosus: a rare case report

**DOI:** 10.11604/pamj.2021.38.377.28762

**Published:** 2021-04-19

**Authors:** Nada Belhajsalah, Marwa Ben Brahim, Syrine Daada, Hajer Ben Brahim, Sonia Hammemi

**Affiliations:** 1 Department of Infectious Disease, Fattouma Bourguiba University Hospital, University of Monastir, Monastir, Tunisia,; 2 Department of Internal Medicine, Fattouma Bourguiba University Hospital, University of Monastir, Monastir, Tunisia,; 3 Laboratory LR12ES05, Lab-NAFS ‘Nutrition - Functional Food and Vascular Health’, Faculty of Medicine, University of Monastir, Monastir, Tunisia

**Keywords:** Mediterranean spotted fever, systemic lupus erythematosus, erythema nodosum, case report

## Abstract

Infections are an important cause of morbidity and mortality in Systemic Lupus Erythematosus (SLE). Mediterranean spotted fever (MSF) is a tick-borne disease caused by Rickettsia conorii. This infection is endemic in Tunisia with summer seasonality. Herein, the case of a 45 years old woman, admitted to hospital with fever and erythema nodosum. On examination, she had a diffuse skin rash, malar rash, and polyarthritis. Serology demonstrated Rickettsia Conoriiinfection. The diagnosis of MSF was made and the patient had a course of doxycycline for 5 days with a prompt improvement of the fever, the skin lesions but she had a persistent malar rash, polyarthritis, and lymphopenia. The immunological profile was positive for antinuclear antibodies (ANA), anti-DNA antibodies, anti-nucleosomes antibodies, and anti-citrullinated protein antibodies (ACPA). The diagnosis of SLE was established. We report the first case of SLE associated with MSF and with erythema nodosum as the initial presentation.

## Introduction

Infections are an important cause of morbidity and mortality in Systemic Lupus Erythematosus (SLE). Mediterranean spotted fever (MSF) is a tick-borne disease caused by *Rickettsia conorii*. This pathogen continues to emerge and reemerge in the Mediterranean region. In fact, MSF is endemic in Tunisia with summer seasonality [1]. MSF is typically characterized by generalized skin rash involving palms and soles, fever, and black eschar [1]. However, atypical forms of MSF including polyarthritis and erythema nodosu0m are more and more reported [2, 3]. SLE may be a predisposing condition to infections due to the impaired cellular and humoral immune functions, and the immunosuppressive treatment prescribed. Some recent studies suggest that certain microbial agents may play a role in the breakdown of immunological tolerance towards host antigens, and the development of immune dysfunction [4]. Herein, we report the first case of MSF association with SLE which can be a trigger, a consequence, or a coincidence.

## Patient and observation

**Figure 1:**
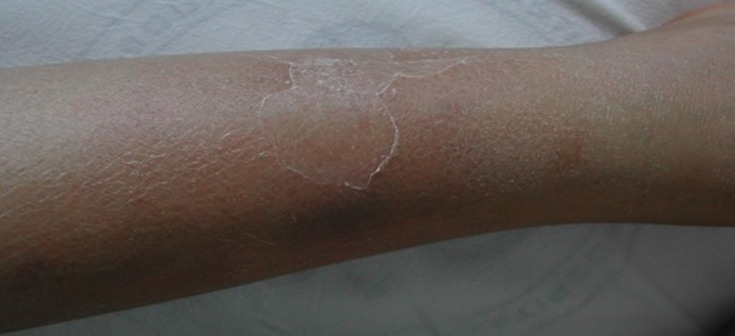
erythema nodosum on lower limbs

**Figure 2:**
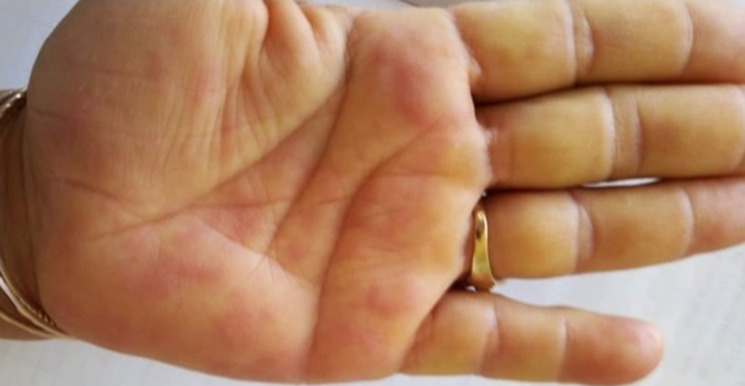
maculopapular rash involving palms

In September, a 45-year-old Tunisian woman, with no personal or familiar medical history, was admitted to internal medicine unit for fever associated with a bilateral lower extremity erythematous rash with an edema extending from the knees to the ankles, evolving over two weeks. Initial examination showed multiple tender nodules consistent with the diagnosis of erythema nodosum (Figure 1). Further examination revealed maculopapular generalized rash involving palms and soles (Figure 2), malar rash, and arthritis of the knees, the ankles, the wrists and the proximal interphalangeal joints. Blood pressure was 120/70 mmHg and pulse rate 80/min. Complete blood count (CBC) revealed normocytic anemia, lymphopenia, and no thrombopenia.

Further lab testing showed accelerated sedimentation rate (110mm/h), and elevated C-reactive protein (73mg/dl). Blood tests for urea, creatinine, electrolytes, and liver function did not reveal any abnormality. Serological set of bacterial and viral causes yielded positive for *Rickettsia conorii. Rickettsia conorii* serology by indirect immunofluorescence showed positivity of immunoglobulin G at 1: 200, and immunoglobulin M at 1: 100. Computed tomography (CT) scan was normal. Subsequently, the diagnosis of MSF was made. She was initially treated with colchicines, and had a course of doxycycline 200 mg for 5 days on an empirical basis. There was a prompt improvement with recovery of the fever, the maculopapular rash, and the erythema nodosum within a week. However, despite treatment, the patient had persistent malar rash, polyarthritis, and lymphopenia. The diagnosis of SLE was established since the immunological profile was positive for antinuclear antibodies (ANA), anti-DNA antibodies, anti-nucleosomes antibodies, and anti-citrullinated protein antibodies (ACPA). X-ray radiography of both hands and feet were normal. The patient received Hydroxychloroquine (5mg/kg/day), and a low dose of prednisone (10mg daily) with clinical improvement of both cutaneous and articular symptoms.

## Discussion

Systemic Lupus Erythematosus (SLE) is an autoimmune disease with multi systemic involvement. This disease has several clinical presentations ranging from mild cutaneous manifestations to sever multiorgan involvement. Our patient had malar rash, arthritis together with lymphopenia, positive ANA, and anti-DNA antibodies. She did fulfill the criteria for SLE. Erythema nodosum is a very uncommon manifestation of SLE. As far as we know, it may be the second reported case in literature, of a SLE inaugurated by an erythema nodosum. In fact, erythema nodosum is the most common clinical form of panniculitis [5]. It is commonly associated with infectious diseases [5]. Streptococcal upper airways infections and tuberculosis are the most frequent infections associated with erythema nodosum [5]. However, very few cases of rickettsioses presenting as arthritis and erythema nodosum have been reported in the literature to date [2, 3].

The association between SLE and infection is well known. Mediterranean spotted fever (MSF) is an acute febrile, zoonotic disease caused by *Rickettsia conorii* and is transmitted to humans by the bites of arthropods. Clinical illness in Rickettsial infection is often non-specific and difficult to diagnose [6]. The most common clinical manifestations are fever, widespread rash involving palms and soles, inoculation eschar, and generalized myalgia and arthralgia [1, 6]. Although considered benign, MSF can lead to serious and potentially fatal forms, with a mortality rate that could reach 2.5% [1]. Identified risk factors for severe forms are: delay of the treatment, old age, and underlying diseases such as diabetes mellitus, cardiac disease, chronic alcoholism, and glucose-6-phophate dehydrogenase deficiency [7]. Although, our patient was predisposed to develop a severe form of MSF, she had a mild form of the disease with a rapidly favorable evolution under anti-Rickettsial treatment.

In clinical practice, clinical suspicion with a positive serology can make the diagnosis of Rickettsial infection [6]. Our patient had fever and maculopapular rash involving palms, and soles associated with a positive *Rickettsia conorii* serology, so the diagnosis of MSF was established. When MSF is suspected or considered, prompt effective antibiotic should be administered. The gold-standard treatment of MSF is doxycycline (200mg daily) [1]. Other classes of antibiotics are effective against *Rickettsia Conorii* such as macrolides, chloramphenicol, and fluoroquinolones [1]. In the present case, defervescence, and improvement of the rash after doxycycline treatment made our diagnosis more evident.

Since MSF is endemic in the Mediterranean, and particularly in Tunisia its occurrence in SLE patients is foreseeable. In fact, the current understanding of the physiopathology of SLE suggests that it results from the interaction between the immune system, hormones, environmental factors, genetic susceptibility, and epigenetic modifications. Those factors do not only make patients more susceptible to infections, but also, there are a number of reports on several viruses, bacteria, and protozoa who are involved in immune dysfunction by molecular mimicry, epitope spreading, and bystander activation [4]. All types of infections, including bacterial, viral, and opportunistic infections, have been reported in patients with SLE. The most common type of infection in lupus patients is bacterial, accounting for 80% of all infections in lupus, followed by viral infections [4]. In fact, SLE patients have an impaired immune function including decreased delayed-type hypersensitivity, reduced numbers of T cells, reduced levels of complement, and functional asplenism [8]. Immunity against *Rickettsia conorii*, who is an obligatory intracellular gram-negative bacterium, implicates humoral and cellular immunity [9]. So in summer time, the abnormality of immune functioning may contribute to an increased risk of MSF in SLE.

## Conclusion

We report the first case of SLE associated with MSF and with erythema nodosum as the initial presentation. Our patient had an atypical presentation of MSF. This infection did inaugurate the onset of SLE. Since there are a number of reports on several viruses, bacteria, and protozoa who are involved in immune dysfunction leading to SLE onset, further investigations are needed to know if *Rickettsia conorii* could be implicated in SLE pathogenesis.
